# Worsening Cognitive Impairment and Neurodegenerative Pathology Progressively Increase Risk for Delirium

**DOI:** 10.1016/j.jagp.2014.08.005

**Published:** 2015-04

**Authors:** Daniel H.J. Davis, Donal T. Skelly, Carol Murray, Edel Hennessy, Jordan Bowen, Samuel Norton, Carol Brayne, Terhi Rahkonen, Raimo Sulkava, David J. Sanderson, J. Nicholas Rawlins, David M. Bannerman, Alasdair M.J. MacLullich, Colm Cunningham

**Affiliations:** a Trinity College Institute of Neuroscience, School of Biochemistry and Immunology, Trinity College Dublin, Republic of Ireland; b Department of Public Health and Primary Care, University of Cambridge, Cambridge, United Kingdom; c Centre for Cognitive Ageing and Cognitive Epidemiology, University of Edinburgh, Edinburgh, United Kingdom; d Department of Clinical Geratology, John Radcliffe Hospital, Oxford, United Kingdom; e Department of Psychology, King's College, London, United Kingdom; f Department of Geriatrics, Jämsä District Municipal Federation of Health Care, Jämsä, Finland; g School of Public Health and Clinical Nutrition, University of Eastern Finland, Kuopio, Finland; h Deparment of Psychology, Durham University, Durham, United Kingdom; i Department of Experimental Psychology, University of Oxford, United Kingdom; j Edinburgh Delirium Research Group, Geriatric Medicine Unit, Edinburgh New Royal Infirmary, Edinburgh, United Kingdom

**Keywords:** Delirium, dementia, neurodegeneration, neuropathology, synaptic, axonal, thalamus, hippocampus, basal forebrain, ageing, cognitive decline, systemic, inflammation, susceptibility, neuroinflammation

## Abstract

**Background:**

Delirium is a profound neuropsychiatric disturbance precipitated by acute illness. Although dementia is the major risk factor this has typically been considered a binary quantity (i.e., cognitively impaired versus cognitively normal) with respect to delirium risk. We used humans and mice to address the hypothesis that the severity of underlying neurodegenerative changes and/or cognitive impairment progressively alters delirium risk.

**Methods:**

Humans in a population-based longitudinal study, Vantaa 85+, were followed for incident delirium. Odds for reporting delirium at follow-up (outcome) were modeled using random-effects logistic regression, where prior cognitive impairment measured by Mini-Mental State Exam (MMSE) (exposure) was considered. To address whether underlying neurodegenerative pathology increased susceptibility to acute cognitive change, mice at three stages of neurodegenerative disease progression (ME7 model of neurodegeneration: controls, 12 weeks, and 16 weeks) were assessed for acute cognitive dysfunction upon systemic inflammation induced by bacterial lipopolysaccharide (LPS; 100 μg/kg). Synaptic and axonal correlates of susceptibility to acute dysfunction were assessed using immunohistochemistry.

**Results:**

In the Vantaa cohort, 465 persons (88.4 ± 2.8 years) completed MMSE at baseline. For every MMSE point lost, risk of incident delirium increased by 5% (p = 0.02). LPS precipitated severe and fluctuating cognitive deficits in 16-week ME7 mice but lower incidence or no deficits in 12-week ME7 and controls, respectively. This was associated with progressive thalamic synaptic loss and axonal pathology.

**Conclusion:**

A human population-based cohort with graded severity of existing cognitive impairment and a mouse model with progressing neurodegeneration both indicate that the risk of delirium increases with greater severity of pre-existing cognitive impairment and neuropathology.

Delirium is a severe neuropsychiatric syndrome characterized by acute cognitive deficits and inattention arising as a consequence of generalized illness.^1^, ^2^ It affects 10%–31% of older hospitalized patients.^3^ Even higher prevalence has been reported in settings associated with frailty (e.g., nursing homes) or critical illness (e.g., intensive care units).^4^ As well as being profoundly distressing for patients, relatives, and care staff,^5^ delirium is associated with multiple poor outcomes: higher mortality, longer hospital stay, and increased institutionalization.^6^, ^7^

Dementia is a strong risk factor for developing delirium,^8^ but the pathophysiology of this relationship is not well established. Part of the difficulty in investigating this in clinical samples is disentangling biological and neuropsychiatric constructs related to delirium (i.e., the acute precipitating disturbance) from the underlying dementia (i.e., the chronic predisposition). Hospital studies have usually relied on duration of dementia diagnosis^9^ or informant scales (e.g., Informant Questionnaire on Cognitive Decline in the Elderly or informant component of the Clinical Dementia Rating Scale) as ways of quantifying pre-existing cognitive deficits.^10^, ^11^ Though these studies have reported that delirium was more likely in persons with apparently more severe prior cognitive impairments, it is difficult to be conclusive about the reliability of such retrospective measures. One prospective study in hospitalized patients showed that severity of dementia progressively increases delirium risk.^12^

Separately capturing pre-existing cognitive function from incident delirium is heuristically (and probably mechanistically) important, but the ability to do this is limited in hospital samples for the reasons outlined above. Here, we present two different approaches (with different strengths and limitations) that together may offer new perspectives. Firstly, we use observational data from an epidemiologic cohort study, where the risk of incident delirium can be more reliably related to baseline cognitive function. This generates hypotheses that can then be tested in an experimental mouse model in which pre-existing cognition and pathology can be controlled more precisely. Interrogation of the contribution of specific features of neurodegenerative pathology to cognitive frailty may add to understanding the basis of delirium risk associated with prior cognitive impairment. Although neither analytic method can directly demonstrate causation, there may be an argument for a degree of coherence between the two approaches.

We hypothesized that severity of pre-existing brain dysfunction progressively increases delirium risk and wanted to investigate this prospectively. We approached this by considering older humans from a population-based cohort^13^, ^14^ who had been assessed with the Mini-Mental State Examination (MMSE).^15^ In parallel, we tested the hypothesis that severity of underlying neurodegenerative pathology would predispose to acute cognitive deficits using mice with none, intermediate, or severe neurodegenerative pathology upon challenging them with systemic inflammation or vehicle control (Table 1). We predicted increased susceptibility to acute dysfunction even before disease-associated cognitive impairment had emerged. We assessed the incidence of delirium in humans and delirium-like cognitive dysfunction in mice at follow-up.

**Table 1:** Comparison of Animal and Population Study Designs

	Mouse (N)	Population Samples (N)
Group	NBH (46)	MMSE score, range 0–30 (468)
	ME7 12w (56)	
	ME7 16w (95)	
Components of outcome assessed	Acute change, fluctuation	Acute change, fluctuation
	Impaired working memory/attention	Inattention, cognition
	Induced by LPS	General medical precipitant

*Notes:* NBH: normal brain homogenate control mice; ME7: prion; MMSE: Mini-Mental State Examination.

The findings of this investigation would provide important information on severity of cognitive decline as a graded risk factor in a true representative elderly population and a possible validation of a small animal model for delirium pathophysiology research. The overall purpose of these analyses is to broaden the methods available for addressing the clinical problem of predisposing cognitive impairment and its relationship to delirium risk.

## Methods

See the Supplementary Appendix (available online) for complete description of the methods.

### Epidemiological Model (Human)

The Vantaa 85+ Study methods have been reported previously.^16^ All individuals aged 85 years residing in the city of Vantaa were invited to participate, with 553 persons recruited (92% of those eligible). Cognitive function was simultaneously assessed by two neurologists at baseline and at four follow-up waves (3, 5, 8, and 10 years). Dementia diagnoses were based on the *Diagnostic and Statistical Manual of Mental Disorders, Third edition, Revised* (DSM-III-R).^17^

The method for incident delirium ascertainment has been described.^13^, ^14^ At each Vantaa interview, the examining neurologists assessed participants and informant(s) for a history of any episodes of delirium, with reference to a checklist of DSM-III-R criteria for delirium diagnosis. The reported history and number of episodes of delirium were corroborated with hospital case notes that were available at the time of assessment. Accordingly, delirium history was retrospectively derived from multiple sources, and the overall diagnosis accepted if the examining neurologists judged that there was sufficient evidence from participant and informant recall and/or indication in the medical notes.

### Experimental Mouse (Model)

An experimental mouse model was used to test the hypothesis that more severe underlying pathology would progressively increase the risk of acute cognitive impairment upon systemic inflammatory challenge. The ME7 prion mouse model is a well-established model of chronic neurodegeneration that leads to progressive synaptic loss, amyloid deposition, microgliosis, and robust neuronal loss with learning and memory deficits.^18^, ^19^ The three experimental groups were as follows: mice injected with: (1) sham control substance (normal brain homogenate; NBH); (2) prion-infected brain homogenate (ME7 strain) surviving for 12 weeks to induce relatively selective hippocampal synaptic loss; or (3) surviving for 16 weeks to induce severe hippocampal and thalamic synaptic/neurodegenerative pathology. These three categories (NBH, 12w ME7, 16w ME7) represent ordered grades of pre-existing neuropathology, but significantly all precede robust neuronal loss and major cognitive impairment. Each group was then injected intraperitoneally with either: (1) a low dose of lipopolysaccharide (LPS, 100 μg/kg), to mimic Gram-negative bacterial infection; or (2) sterile saline, as a control. All experiments were performed in compliance with the Cruelty to Animals Act (1876) and the European Community Directive, 86/609/EEC.

#### Murine Cognitive Function

Cognitive function was assessed using T-maze alternation. This task, which requires attention to the maze arm initially visited, retention of this information for the 25-second intra-trial interval, and execution of the opposite turn to escape upon re-exposure to the maze, has been described in detail.^20^ Deficits on this task may reflect inability to modulate the amount of attention paid to recently experienced stimuli^21^, ^22^ and thus could potentially be related to the acute, fluctuating inattention and other cognitive deficits seen in delirium precipitated by a general medical condition (Table 1). Animals were trained (10 trials daily) and those achieving a criterion of 70% or greater alternation for 2 or more consecutive days were challenged with LPS or saline. Animals were tested every 20 minutes (3–9 hours post-LPS, 15 trials). Experiments were performed in Oxford and Dublin, by four different blinded experimenters to ensure robustness and reproducibility. Chance responding in this maze is 50% alternation and “acute impairment” was defined as 3 out of 5 correct in two or more blocks or 2 or fewer out of 5 correct in any one block. Differences in incidence, so defined, between groups were assessed using Fisher's exact test.

#### Neuropathology

Mouse brains were wax-embedded and sectioned (10 μm). To demonstrate the relevant neuropathology, these were labeled with antibodies against synaptophysin (synaptic density marker) and neurofilament heavy chain and amyloid precursor protein (axonal damage markers). Sections were photographed and synaptic density and axonal pathology were recorded in the hippocampus and posterior nuclei of the thalamus. Antibody labelling was quantified by standardized methods (detailed in Supplementary Material; available online).

### Statistical Analyses

#### Epidemiology

Odds for reporting delirium at follow-up (outcome) were modeled using random-effects logistic regression, adjusting for age, sex, and co-morbidity score based on the Charlson Index.^23^ Participants assessed in multiple waves were able to contribute to the model for each observation, and robust standard errors were estimated to account for the clustered nature of these data. Time-in-study was used as the time metric and covariance matrices were unstructured.

In the majority of studies, the analytic properties of the MMSE are often limited by ceiling effects that result in highly skewed distributions. In our population of older individuals (mean age 88 years), however, the mean MMSE score (19.9) is very close to the median (21), so the assumptions hold for the Gaussian distribution required for modeling the MMSE as a continuous parameter (Supplementary Fig. 1; available online). Formal post-estimation tests of goodness-of-fit (Hosmer-Lemeshow) were applied to check any violation of assumptions.

#### Experimental Model

##### Cognitive function

The number of correct turns, scored over five trials, was considered as count data for each time point during the experiment, with a random-intercept multilevel Poisson model with indicator variables fitted for each time point. The number of correct turns was specified as the outcome, and experimental category (NBH, 12w ME7, 16w ME7) and challenge (saline or LPS) as exposures along with any interactions. The time points compared were between baseline, and challenge (3–5, 5–7, 7–9 hours) and recovery (22, 24 hours) periods, respectively. The reference category was the average performance of NBH mice at baseline (i.e., −24 and −22 hours before LPS or saline), allowing comparisons to be made both within and between groups. Fluctuation about the mean performance post-acute challenge was calculated for each individual animal. A score of 60, 80, 60 (mean: 66.7) is given an index of 6.7 + 13.3 + 6.7 = 26.7, and 40, 80, 60 (mean: 60) is given 20 + 20 + 0 = 40. Differences in fluctuation between experimental groups were assessed using the Mann-Whitney test.

##### Pathology

Synaptophysin density and axonal varicosities in ME7 and NBH animals (12 and 16 weeks) were compared by one-way analysis of variance (ANOVA) with Bonferroni corrections for post hoc pairwise comparisons.

## Results

### Epidemiology

At baseline, 465 (84%) individuals had a completed MMSE. Mean MMSE was 19.9 (SD: 7.0) points (normal distributions shown in Supplementary Fig. 1; available online), and mean age was 88.4 years (SD: 2.8). Women made up 364 of 465 (78%) of the cohort. Follow-up data on delirium incidence was available in 272, 153, 49, and 19 persons at 3, 5, 8, and 10 years, respectively.

**Figure 1:**
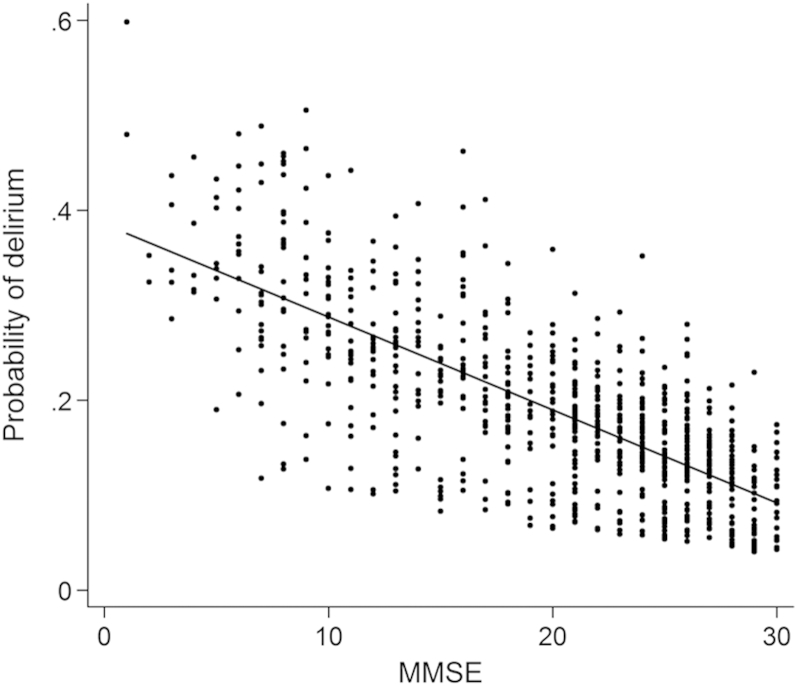
Delirium risk in relation to prior MMSE. The probability of delirium is plotted against previous MMSE score, where each point is an observation of delirium risk at follow-up. The model-predicted coefficient for delirium risk is described as a straight line with a negative slope, showing an inverse relationship.

In total, 81 episodes of delirium were recorded at follow-up. Table 2 reports the odds of incident delirium as a function of baseline cognition; Figure 1 shows the probability of delirium over the range of observed MMSE scores. For every MMSE point lost, the risk of incident delirium increased by 5% (p = 0.02). For example, the probability of incident delirium at follow-up for an 85-yearold man with MMSE = 28 points at baseline would be 0.12, rising to 0.29 for an equivalent individual with an MMSE score of 10 points at baseline. Co-morbidity score did not influence likelihood of incident delirium (p = 0.63). Goodness-of-fit testing showed no problems with model calibration.

**Table 2:** Logistic Regression Model Showing Odds of Incident Delirium in Relation to Baseline Cognitive Function

	OR	95% CI	p Value*
MMSE, per point	0.95	(0.92–0.99)	0.018
Age, per year	1.08	(1.00–1.18)	0.056
Sex, women cf. men	2.36	(1.03–5.40)	0.037
Comorbidity score	1.04	(0.90–1.19)	0.636

*Notes:* N = 468 in 257 clusters. Goodness of fit for model residuals p = 0.053 (Pearson χ^2^ = 426.77). *2-tailed p value used in testing the null hypothesis that the coefficient (parameter) is 0, Wald χ^2^ for 4 degrees of freedom = 20.72. MMSE: Mini-Mental Status Examination.

Random-effects models account for data missing at random. However, the 16% of baseline assessments with incomplete MMSE are likely to be non-random. Indeed, previous analyses showed this group to have a risk of delirium similar to those with MMSE scores in range less than 22 points (data not shown). Nonetheless, a sensitivity analysis, whereby missing MMSE scores were assigned an imputed value of 0 did not significantly change the estimated parameters or conclusions (Table 3).

**Table 3:** The Effect of Missing MMSE at Baseline (Unclassifiable Participants) on Delirium Outcomes

	OR	95% CI	p value*
MMSE, per point	0.965	(0.933–0.998)	0.036
Age, per year	1.073	(0.988–1.167)	0.093
Sex, women cf. men	2.211	(1.003–4.877)	0.049
Comorbidity score	1.029	(0.893–1.185)	0.693

*Notes:* Logistic regression model showing odds of incident delirium according to baseline cognitive function, including participants with incomplete MMSE assessments, where this was imputed as lowest score (MMSE = 0). N = 492 in 237 clusters. Goodness of fit for model residuals p = 0.085 (Pearson χ^2^ = 444.55). *2-tailed p value used in testing the null hypothesis that the coefficient (parameter) is 0, Wald χ^2^ for 4 degrees of freedom = 17.97. MMSE: Mini-Mental Status Examination.

### Experimental Model

#### Acute Cognitive Dysfunction in Mice

**Figure 2:**
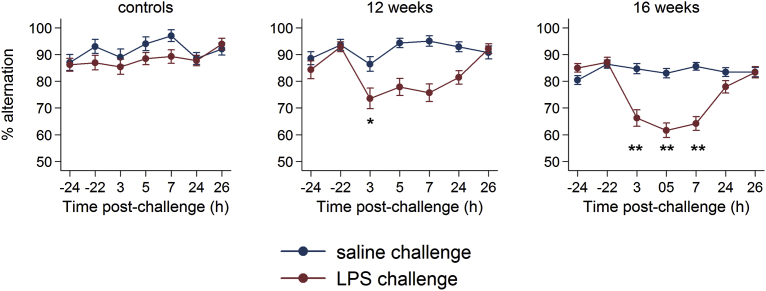
Acute cognitive impairments induced by LPS are more severe in animals with more advanced neurodegenerative disease. Performance of NBH (normal) mice, ME7 animals 12 weeks post-inoculation, or ME7 animals 16 weeks post-inoculation upon challenge with saline or LPS (100 μg/kg), plotted with intervals showing the standard error of the mean. Criterion baseline performance (≥70%) was ascertained for all challenged animals, and these were then tested 15 times (every 20 minutes for >5 hours: 3h–9h post LPS) post-challenge as well as for 10 further trials 24 hours post-challenge. Statistically significantly different RR for errors are denoted by *p <0.05 and **p <0.01.

Animals challenged with LPS display significantly lower arousal and marked hypoactivity (Supplementary Video 1; available online). Figure 2 shows performance of NBH and 12w and 16w ME7 mice over time on the T-maze alternation task during LPS or saline challenge. Although still greater than or equal to 80%, baseline performance was significantly lower in 16w ME7 than in NBH mice (relative risk [RR] for errors: 2.11, 95% confidence interval [CI] 1.43 to 3.13, p <0.01, Supplementary Tables 1, 2; available online). ME7 12w were not significantly cognitively impaired with respect to NBH mice. We hypothesized that susceptibility to an acute stressor would increase with progressing disease (i.e., 16w > 12w > NBH). Testing an interaction between LPS challenge and disease stage demonstrated a difference in magnitude of LPS effect in each of the three categories (p <0.01, Supplementary Table 2; available online).

Defining a deficit as less than or equal to 60% alternation (i.e., ≤3/5 correct trials) in at least two blocks on the treatment day or less than or equal to 40% (≤2/5 correct trials) in any one block, LPS produces impairment in fewer than 4% of normal animals, in 36% of 12w animals, and in 77% of 16w animals. Thus, incidence of acute deficits increases with progressing underlying pathology (Fisher's exact test, p <0.01). Importantly, susceptibility to acute disruption was present before baseline cognitive differences emerged (i.e., at 12w).

When challenged with LPS there was no deficit in normal (NBH) mice compared with saline challenge (errors at 3 hrs RR: 1.1 [p = 0.74]; at 5 hrs RR: 0.8 [p = 0.35], at 7hrs RR: 0.6 [p = 0.12], Supplementary Table 3; available online). The same LPS challenge produced a transient deficit in function in 12-week ME7 mice (errors at 3 hrs RR: 1.8 [p = 0.02], recovering by 5 hrs) whereas a prolonged and increased deficit is seen in 16-week ME7 mice (errors at 3 hrs RR: 2.1 [p <0.01]; at 5 hrs RR: 2.4 [p <0.01]; at 7 hrs RR 2.2 [p <0.01], Supplementary Table 5; available online).

**Figure 3:**
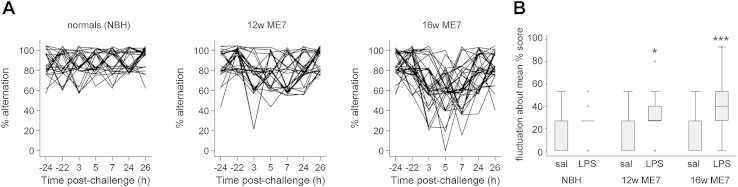
Systemic inflammation induces fluctuating cognitive deficits only in animals with existing neurodegenerative disease. [A] Individual trajectories of performance in mice given LPS challenge, given in each experimental group. Plots are jittered on the *y*-axis to distinguish overlapping plots. [B] Fluctuation about the mean correct performance (% trials) according to experimental group. NBH normal brain homogenate (controls), 12w ME7 mice, and 16w ME7 mice. Mann-Whitney: NBH-LPS versus NBH-saline, p = 0.16; ME7 12w-saline versus ME7 12w-LPS, p = 0.03 (denoted *); ME7 16w saline versus ME7 16w-LPS, p <0.001 (denoted ***).

The trajectory of each individual LPS-treated animal is shown in Figure 3, according to experimental category. Performance in NBH was generally over 80% and decreases to 60% were not sustained for more than one block. Impairments were more severe and sustained in ME7 mice. Troughs in T-maze performance were most evident in 16w ME7 mice and could occur in any or all of the post-LPS trial blocks. Therefore cognitive dysfunction fluctuates with time. Figure 3B shows the median fluctuation about the mean, which represents the distance from the mean post-LPS performance of each individual animal for each block of 5 trials post-challenge (see methods). LPS induced statistically significant fluctuation increases in 12w ME7 animals (Mann-Whitney, z = −2.22, p = 0.03) and 16w ME7 animals (z = −4.02, p <0.001), but not in NBH animals (z = −1.4 p = 0.16).

#### Neuropathological Changes Underpinning Increased Risk

**Figure 4:**
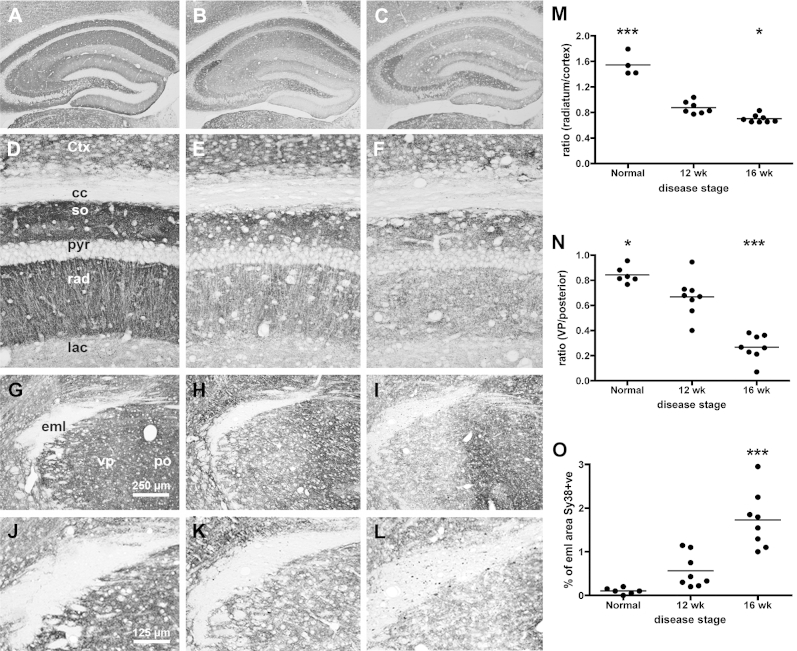
Synaptic integrity deteriorates as a function of disease progression. Formalin fixed, paraffin-embedded brains from NBH and ME7 animals, at 12 and 16 weeks post-inoculation, were sectioned (10 μm) and labelled with antibodies against synaptophysin (Sy38). [A–C] Gross view of hippocampus of normal, 12w, and 16w, showing severe synaptic loss in the strata of the CA1 and dentate gyrus, but preservation of the DG -> CA3 projection. [D–F] Higher power pictures of these strata, used for quantification in the hippocampus (M) according to the equation T_cc_ − T_rad_/T_cc_ − T_Ctx_ (G–I; see Supplementary Methods; available online) 10x pictures of the thalamus at approximately bregma −2.5 (AP), showing the external medullary lamina (eml), ventroposterior nucleus (VP) and the posterior nucleus (Po) and clear synaptic loss in the VP at 16 weeks. [J–L] Higher power pictures of these areas, used for quantification of thalamic synaptic density according to the equation T_eml_ − T_VP_/T_eml_ − T_Po_ (N; see Supplementary Methods; available online) and % sy38-positive area within the eml (O). Pairwise comparisons to ME7 12 week animals by Bonferroni post-hoc tests after significant main effect of disease stage by one-way ANOVA. *p <0.05, ***p <0.001.

Synaptic loss is a strong correlate of cognitive decline in dementia.^24^, ^25^ Synaptic density in the hippocampus (Fig. 4A–C and, higher power, D–F) decreases in prion-diseased mice at 12 weeks (Fig. 4B) and 16 weeks (Fig. 4C) post-inoculation compared with normal mice (Fig. 4A). The ratio between density in the stratum radiatum and the neocortex is shown in Figure 4M. Thus, synaptic integrity decreases with disease progression (one-way ANOVA, effect of disease stage: F_(2,16)_ = 86.54, p <0.0001) with significantly lower synaptic density in 12w than NBH (p <0.001), and a smaller further decrease at 16w (p <0.05).

There was significant thalamic synaptic loss in ME7 mice, particularly in the ventroposterior nuclei when compared with the posterior nucleus. Regarding the synaptic density ratio between these two structures (4n), a one-way ANOVA showed an effect of disease stage (F_(2,19)_ = 45.75, p <0.0001). This synaptic loss was relatively mild at 12w (12w versus NBH, p <0.05; Fig. 4H), but significantly worse at 16w than at 12w (p <0.001; Fig. 4I). Furthermore, in 16w ME7 animals the external medullary lamina, an interthalamic white matter tract, showed many large spheroidal synaptophysin deposits (Fig. 4I), representing significant axonal/white matter pathology. One-way ANOVA (Fig. 4O) showed a main effect of disease stage (F_(2,19)_ = 24.12, p <0.0001) and significant increase at 16w with respect to both NBH and 12w animals (p <0.001). This axonal pathology was not present at 12w. Similar white matter synaptophysin deposits were observed in the fimbria (axons from basal forebrain cholinergic areas to the hippocampus), and in the internal capsule (thalamic output to the cortical mantle) (Supplementary Fig. 2; available online). Therefore, susceptibility to robust cognitive dysfunction associates with the spread of pathology to the thalamus and to multiple white matter tracts.

**Figure 5:**
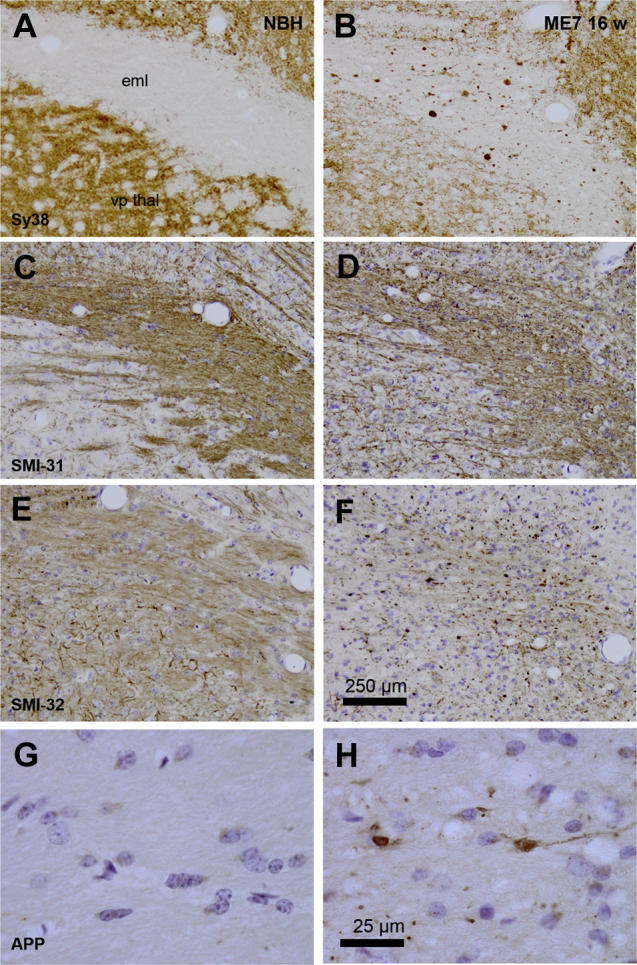
Axonal pathology in animals at 16 weeks of disease progression. Formalin fixed, paraffin-embedded brains from NBH and ME7 animals, 16 weeks post-inoculation, were sectioned (10 μm) and labelled with antibodies against synaptophysin (Sy38 [A,B]), phosphorylated neurofilament heavy chain (SMI-31 [C,D]); unphosphorylated neurofilament heavy chain (SMI-32 [E,F]) and amyloid precursor protein (APP695 [G,H]). Photograph with 10x objectives, except [G,H]: photograph under 100x oil immersion objective. Scale bar for [A–F] shown in [F] = 250 μm, and that in [H] serves for panels [G] and [H] (25 μm). vp thal: ventroposterior thalamic nucleus; eml: external medullary lamina.

Because altered axon morphology shows strong correlation with dementia status,^26^ we sought further evidence for axonal pathology in 16w ME7 animals. We labeled the thalamus with antibodies for axonal pathology (Fig. 5) in NBH and ME7 16w animals. Synaptic element accumulation in the external medullary lamina white matter (Fig. 5B) was associated with discontinuity of axons (Fig. 5D), loss of elongated neurofilament labeling and the appearance of spheroids/varicosities (Fig. 5F). Axonal pathology was also demonstrated by APP-positive axonal spheroids in the external medullary lamina (Fig. 5H). Thus, there were graded levels of synaptic and axonal pathology in ME7 animals, with the relationship 16w > 12w > NBH.

## Discussion

This study has demonstrated a strong association between severity of existing cognitive impairment and future delirium risk in a true population-based sample of older humans, recapitulated in a mouse model of acute and fluctuating cognitive dysfunction.

### Prior Cognitive Impairment as a Risk Factor for Delirium

It is established that dementia or prior cognitive impairment is a significant risk factor for delirium.^8^ This risk factor has typically been dichotomized, however, such that patients are designated either cognitively impaired or cognitively normal. A strength of the current study is that subjects were assessed in respect of a continuum of cognitive function (in the case of humans) or graded categories of neurodegenerative pathology (in the case of mice). Both data sets demonstrate increasing susceptibility to acute deficits with increasing underlying cognitive or neurodegenerative pathology, providing empirical human and animal evidence that dementia-associated risk is not binary but rather is on a continuum. The use of MMSE rather than more comprehensive cognitive testing was a limitation, although scores were normally distributed and no ceiling effect was observed. Retrospective delirium ascertainment in Vantaa 85+ is a further limitation of the study. This is mitigated to an extent, however, by corroboration from multiple sources including hospital and primary care notes. Medical records have been validated for the diagnosis of delirium history,^27^ and the diagnostic accuracy for past episodes is likely to be higher if case notes are reviewed in conjunction with clinical interview, as is the case here. Missing data on MMSE at baseline was informative; here it was associated with higher delirium risk. This is consistent with other observations that inability to complete cognitive testing is associated with poorer outcomes.^28^, ^29^ That the comorbidity burden does not appear to be significantly associated with delirium risk is in keeping with data showing that the association between delirium and adverse outcomes is over and above that expected for comorbidity burden alone.^30^ Nevertheless, it is difficult to be definitive here as the present analysis lacks power to fully explore the effects of frailty and comorbidity on delirium and dementia.

### Refinement and Validation of a Mouse Model of Delirium During Dementia

For the first time in any animal model of neurodegeneration, we have demonstrated progressively increased risk of acute cognitive dysfunction upon application of a standardized systemic inflammatory insult in animals with advancing synaptic and white matter pathology. Applying DSM-IV criteria for delirium, the mouse model demonstrated an acute onset, fluctuating, change in cognition not better accounted for by dementia, induced by LPS (a reasonable mimic of a general medical condition effecting physiological disturbance). The nature of cognitive/attentional impairment requires discussion. The T-maze task used here relies on working memory^31^ and an ability to focus and shift attention between arms of the maze. Performance is proposed to reflect short-term reduction in attention to recently visited spatial locations.^21^, ^22^ Thus animals either fail to attend to the location they are currently visiting on the sample run, or fail to remember or habituate to that location just 25 seconds later. This study is also the first, to our knowledge, to demonstrate fluctuating course in inflammation-induced cognitive dysfunction. In addition, LPS-treated ME7 animals show exaggerated hypoactivity (psychomotor disturbance), significantly reduced interaction with their environment (Supplementary Video 1; available online) and impaired acquisition of new spatial memories in a visuospatial Y-maze escape task,^32^ but preservation of long-term visuospatial memory if acquired prior to LPS treatment. These data, therefore, show psychomotor disturbance and fluctuating impairment of dynamic cognitive processes and may be consistent with human delirium data showing impairments on cognitive tasks involving online processing of novel, trial specific, information but preservation of previously acquired long-term memories.^33^ We believe that the dysfunction occurring in this mouse model may fulfill the DSM-IV criteria adequately (Table 1) and given the constraints of examining cognitive function in markedly hypoactive animals, this T-maze task is appropriate for interrogating an individual's ability to attend to and process cues from its environment to inform behavior. Our results are also consistent with recent data showing impaired reversal learning and attention after systemic inflammatory insult.^34^

The current mouse model mirrors the human epidemiological finding that greater severity of disease confers progressively increased risk for delirium. This represents a significant validation of this animal model, indicating that it has predictive value and reinforcing the impetus to use findings arising from this model to explore aspects of delirium pathophysiology. It is important to stress that heterogeneous clinical presentations cannot be modeled by a single experimental system. The model recapitulates hypoactivity, altered arousal and fluctuating cognitive deficits in domains relevant to delirium and as such addresses aspects of hypoactive delirium triggered by systemic inflammation superimposed on existing dementia. Generalizations beyond this should be treated cautiously.

### Delirium Pathophysiology

We demonstrated an effect of severity of neuropathology on risk of acute and fluctuating cognitive deficits. The neuropathological features shown here may be directly relevant to delirium pathophysiology. Although the neuroanatomy of escape-from-water T-maze alternation has been little studied, the hippocampus is undoubtedly, but not exclusively, involved: there is evidence also for prefrontal cortex, thalamus, and amygdala involvement in impaired spontaneous alternation.^35^, ^36^, ^37^, ^38^ Robust white matter pathology in the fimbria (Supplementary Data; available online), which connects the septum to the hippocampus, and disruption in this T-maze by muscarinic cholinergic receptor antagonism (scopolamine)^39^ suggest roles for the septohippocampal pathway and cholinergic neurons, although robust cognitive dysfunction was more strongly associated with thalamic ventroposteromedial synaptic and white matter pathology (at 16 weeks). The thalamus processes sensory information before output to the cortex via the internal capsule, which is also pathologically affected here (Supplementary Data; available online), and thalamic ischemic lesions are also reportedly associated with delirium.^40^, ^41^ Furthermore key arousal centers of the brain, the locus coeruleus noradrenergic and brainstem acetylcholinergic neurons, have opposite effects on spontaneous activity in the ventroposteromedial thalamus in rodents^42^ with corresponding effects on arousal (cortical activation and deactivation respectively).^43^ Thus, thalamic pathology might underpin the markedly suppressed arousal observed in ME7+LPS mice^32^ (Supplementary Video 1; available online) and perhaps in humans. Although inattention is a core feature of delirium and requires frontal and parietal cortex communication,^44^ this cannot occur without sufficient arousal, which is generated in sub-cortical structures.^45^ It has been reported that altered level of arousal is a strong predictor of inattention and associates with human delirium.^46^ Therefore, it is possible that sub-cortical structures affecting arousal have an important role in hypoactive delirium. Specific evidence for prior cortical pathology as a risk factor is sparse and conflicting: Frontotemporal dementia and early-onset Alzheimer disease (AD) constitute lesser risk factors for delirium than more distributed pathologies like vascular dementia and late-onset AD,^9^ while existing impairment in executive function, which is frontal cortex dependent, selectively predisposes to delirium.^47^, ^48^ Clearly, the neuroanatomical basis of increased delirium risk requires more research.

The progressive loss of presynaptic terminals and the accumulation of white matter pathology shown here represent significant brain disconnectivity and quantifiable loss of “brain reserve”.^49^ Synaptic loss is a strong correlate of cognitive decline^50^ and neuronal pathology occurs many years before cognitive impairment in AD.^51^ The mouse data suggest that prior degenerative pathology increases risk for acute cognitive impairment even before baseline cognitive impairment has emerged and we propose that synaptic loss will predict susceptibility to delirium in humans. Interestingly, in healthy volunteer experiments acute systemic inflammation did not affect performance on the Stroop test of sustained attention/executive function but successful performance under systemic inflammation recruited significantly more distributed brain areas.^52^ Recruitment of additional brain areas during systemic inflammation will likely be hindered by synaptic and axonal pathology such as that demonstrated in the current study and we hypothesize that existing synaptic loss is a major predisposing factor for the precipitation of delirium by mild/moderate infection in advanced age/dementia.

Although synaptic disconnection may be a major contributor to delirium risk, other aspects of dementia, such as microglial activation and chronic hypocholinergic function, are also likely to play significant roles. Both cyclooxygenase-1 inhibition and IL-1 receptor antagonist are protective in this model.^53^ Similarly, cholinergic lesions increase susceptibility to inflammation-induced cognitive deficits^39^ and there is evidence that acetylcholinergic tone modulates inflammatory responses both centrally and peripherally.^54^, ^55^

Finally, delirium increases the risk of subsequent dementia,^13^, ^56^, ^57^ but the current data suggest that delirium unmasks an underlying neurodegenerative process rather than creates this neuropathology outright. The acute deficits triggered in this study were reversible and did not cause additional loss of synaptic terminals but LPS challenges have been deliberately mild here to facilitate cognitive testing. Significantly higher LPS doses do induce significant new pathology and alter disease-associated decline^58^, ^59^, ^60^, ^61^ consistent with observations in the Vantaa 85+ cohort.^13^

## Conclusion

The current data represent the clearest empirical demonstration that greater degrees of neurodegeneration at baseline increase the risk, duration, and severity of delirium. They support the established predisposing/precipitating factor model of delirium,^62^ and answer recent calls to elucidate predisposing factors further.^8^, ^63^, ^64^ We hypothesize that the axonal and synaptic pathology shown here constitute key factors contributing to the overall frailty of brain function and may underpin the failure of the degenerating brain to demonstrate resilience upon “stress-testing” with systemic inflammation. Epidemiological, experimental, and clinical efforts are vital to identify prevention and management strategies of this common, distressing, and serious condition. Animal models may well have utility in unraveling molecular mechanisms.
