# Supplementary material for: Worsening Cognitive Impairment and Neurodegenerative Pathology Progressively Increase Risk for Delirium
Source: Am J Geriatr Psychiatry. 2015 Apr;23(4):403–15. doi: 10.1016/j.jagp.2014.08.005 (PMC4278840; doi:10.1016/j.jagp.2014.08.005)
Supplement: Supplementary Materials [file mmc1.docx]

**Supplementary Methods**

*ME7 prion disease: surgical initiation of disease*

ME7 prion diseased mice show progressive neurodegeneration characterised by extracellular amyloidosis, synaptic loss and cognitive decline similar to clinical dementia in humans, with early pathology primarily seen in the entorhinal cortex, hippocampus and septum ([Betmouni and Perry, 1999](#_ENREF_2), [Cunningham *et al.*, 2003](#_ENREF_8)). They show cognitive deficits that begin to become apparent at between 13 and 17 weeks post-inoculation. Female C57BL/6 mice at 8–10 weeks of age (Harlan Olac Ltd, Bicester, UK) were anaesthetised and bilaterally stereotactically injected with 1 μl of a 10% w/v scrapie (ME7 strain)-infected C57BL/6 brain homogenate (or 10% w/v normal brain homogenate (NBH). Animal experimentation was performed at 2 sites, The Department of Experimental Psychology in the University of Oxford and the Trinity College Institute of Neuroscience, and was carried out under UK Home Office License in the former (PPL30/1989) and under license granted by the Minister for Health and Children, Ireland, with approval from the Trinity College Dublin Animal Research Ethics Committee in the latter (B100/3907). All experiments were performed in compliance with the Cruelty to Animals Act, 1876 and the European Community Directive, 86/609/EEC. Every effort was made to minimise stress to the animals.

*Intraperitoneal challenges*

Separate experimental groups with NBH or ME7 at either 12 or 16 weeks post-inoculation were injected intraperitoneally with 100 µg/kg of lipopolysaccharide (LPS; equine abortus, Sigma L5886, Poole, UK) in a volume of 200 µl saline. This dose mimics a mild infection, producing small changes (≈1°C) in core body temperature. Controls were administered 200 µl non-pyrogenic saline in each case. Since NBH animals have no discernible pathology and show no cognitive decline, NBH animals at 12 weeks and 16 weeks post-inoculation were combined in the statistical analysis and described simply as NBH+saline or NBH+LPS.

*Murine cognitive function: T-maze alternation*

Working memory (3–9 hours post-LPS) was assessed using alternation behaviour in a high-walled, black perspex T-maze, adapted by the addition of water to a depth of 2 cm, in order to motivate animals challenged with LPS to explore and solve the maze. This task has been previously described in detail [[2](#_ENREF_2)]. Each mouse was placed in the start arm of the maze with 1 arm blocked such that they were forced to make a left (or right) turn, selected in a pseudorandom sequence (equal numbers of left and right turns, no more than 2 consecutive runs to the same arm). On making this turn the mouse could escape from the water by entering the small tube, and then a transit tube, in which it was carried to another holding cage. The mouse was held here for 25 seconds (intra-trial interval) during which time the guillotine door of the T-maze was removed and the exit tube was switched to the alternate arm. The mouse was then replaced in the start arm and could choose either arm. The mouse must alternate from its original turn to escape. On choosing correctly mice escape to the transit tube as before and are returned to their home cage. On choosing incorrectly the mice were allowed to self-correct to find the correct exit arm. Animals were trained in this maze (10 trials daily) until consistently achieving ≥70% alternation and their performance was then maintained at this level until 12 or 16 weeks post-inoculation. On the day of acute inflammatory challenge, only animals achieving this criterion for ≥2 consecutive days were challenged with LPS or saline and these animals were tested every 20 minutes for approximately 5 hours post-challenge (therefore 15 times in total on the day of LPS challenge). T-maze performance after LPS challenge was assessed at 2 different sites (Oxford and Dublin) and by 4 different experimenters who were, in all cases, blind to the experimental treatment. Chance responding in this maze is 50% alternation. In a block of 5 trials 60% represents 3 correct trials out of 5 and 40% represents 2 correct trials out of 5. We have thus defined robust impairment as 3/5 in 2 or more blocks on the challenge day, or 2/5 or less in any one block on the challenge day (Differences between groups were assessed using Fisher’s exact test).

*Quantification of neuropathology*

A cohort of ME7 and NBH animals, which were not challenged with LPS, were transcardially perfused with heparinised saline followed by 10% formalin, under terminal anaesthesia with sodium pentobarbital (Euthatal; Merial, Harlow, UK), and then paraffin wax-embedded for microtome sectioning and immunohistochemistry. Sections (10µm) were rehydrated and labelled with antibodies against synaptophysin (Sy38, Millipore, Dublin), amyloid precursor protein (APP: Invitrogen, Bio-Sciences, Dublin) and phosphorylated (SMI-31) and de-phosphorylated (SMI-32) 200kDa neurofilament heavy chain (Covance, gifted by Dr. Fabian Docagne, Caen, France). Briefly, nonspecific peroxidase activity was eliminated by incubating sections in 1 ml H_2_0_2_/100 ml methanol (1% H_2_0_2_) for 10 minutes before antigen retrieval was carried out by heating in 0.2M boric acid (pH 9) for 30 minutes at 60 degrees (Sy38) or 0.01M citrate buffer (pH 6). After washing, sections were blocked with normal sera and incubated overnight with primary antibodies at 1/2000 (Sy38), 1/1000 (SMI-31), 1/200 (SMI-32) or 1/100 (APP). Labelling was completed using a biotinylated secondary antibody, ABC complex and nickel diaminobenzidine (DAB) as a chromogen. Synaptophysin labelling was enhanced by supplementation of the DAB reaction with 0.06% ammonium nickel sulphate.

Tissue sections were photographed using an Olympus DP25 camera and cell A®software (Mason, Dublin) mounted on a Leica DM3000 (Lab instruments and supplies, Ashbourne, Co. Meath). Images were converted to 16 bit files and analysed in ImageJ (NIH, Bethesda). Transmittance (T; lux) was recorded in the hippocampal layers corpus callosum (cc) and stratum radiatum (rad), in overlying cortex (ctx) and in the external medullary lamina (eml), ventroposterior (vp) and posterior (po) nuclei of the thalamus. We have previously identified the hippocampus and thalamus as areas of most severe pathology in this model [[1](#_ENREF_1)]. Ratios were then calculated for the hippocampus according to the equation T_cc_-T_rad_/T_cc_-T_Ctx_ and for the thalamus according to the equation T_eml_-T_VP_/T_eml_-T_Po_. In addition, the % area of the external medullary lamina composed of Sy38-positive elements was calculated using the measure particles function in ImageJ, using a particle size range of 20-infinity and a circularity range of 0.7-1.

***Statistical Analyses***

*Experimental model: T-maze performance*

The number of correct turns, scored over five trials, was considered as count data for each time point during the experiment. To account for changes over time, a random-intercept multilevel Poisson model with indicator variables for each time point was fitted. This has advantages over a traditional ANOVA approach as the latter is designed to model differences in means of equal variances. For continuous data, means and variances have straightforward interpretations. However, interpretation of these quantities is less clear when the outcome is categorical, even if the data are considered as proportions. The random-intercept accounts for intra-individual variation over the course of the experiment, akin to a Bonferroni correction for multiple testing. Therefore, the full model specified the number of correct turns as the outcome, and experimental category (NBH, 12w ME7, 16w ME7) and challenge (saline or LPS) as exposures along with any interactions. The time points compared were between baseline, and challenge (3-5, 5-7, 7-9 hours) and recovery (22, 24 hours) periods respectively, where the reference category is the average performance of NBH mice at baseline (i.e. -24 and -22 hours before LPS or saline). Choosing such a reference category allows comparisons to be made both within and between groups. This model was used to test the following questions:

1. Does the effect of challenge type vary in the different experimental groups?

Tests the effect of a challenge*group interaction on number of correct turns

1. Does the time course during challenge for errors vary in different experimental groups?

Tests the effect of a time point*group interaction on number of correct turns, where time point is from 3, 5, or 7 hours post-challenge

In order to test differences in performance fluctuation due to the effect of LPS between experimental groups, we calculated fluctuation about the mean performance post-acute challenge for each individual animal. For example, a score of 60, 80, 60 (mean = 66.7) would be given an index of 6.7 + 13.3 + 6.7 = 26.7. A score of 40, 80, 60 (mean = 60) would result in an index of 20 + 20 + 0 = 40. Differences in fluctuation were assessed using the Mann-Whitney test.

*Experimental model: pathology*

Comparisons of synaptophysin densities and axonal varicosities in ME7 versus NBH animals (12 and 16 weeks) were performed by one-way analysis of variance (ANOVA) with Bonferroni corrections for post-hoc pairwise comparisons.

***Supplementary results*.**

MMSE Distribution: In the majority of studies, the analytic properties of the MMSE are often limited by ceiling effects that result in highly skewed distributions. However, in this population of older individuals (mean age 88), the mean MMSE score (19.8) is very close to the median (21), so the assumptions hold for the Gaussian distribution required for modeling the MMSE as a continuous parameter (supplementary figure 1). Formal post-estimation tests of goodness-of-fit (Hosmer-Lemeshow) were applied to check any violation of assumptions.


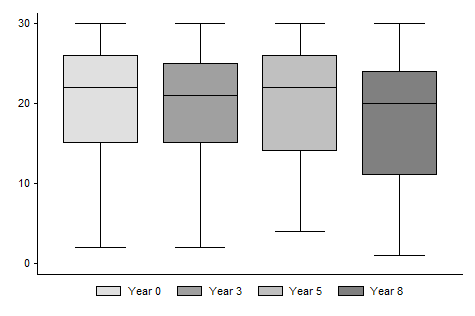


**Supplementary figure 1. Distribution of MMSE scores in population at each examination**. The distribution of MMSE scores is shown at each study wave. Boxes represent the interquartile range and whiskers the 99% distribution. The median is indicated in the middle of each box. Broadly, the MMSE does not show ceiling effects in this population aged ≥85 years.

*Arousal*

ME7 mice challenged with LPS (100 µg/kg) show markedly altered arousal (see additional supplementary video 1: Arousal LPS vs saline.mp4). The 2 animals on the left of the screen can be seen to show significant suppression of activity and low level of engagement with the environment/cage mates, with respect to the saline-treated animal that is seen to explore the cage.

*White matter Pathology*.

Synaptophysin immunohistochemistry was used to demonstrate significant deposition of synaptic protein in white matter tracts. The internal capsule is the major tract for axons connecting the thalamic nuclei to the cortex and in ME7 animals at 16 weeks this tract shows clear evidence of axonal pathology compared to NBH animals, as assessed by the mislocalisation of synaptophysin (Supplementary figure 1). Similarly the fimbria carries axons from the basal forebrain cholinergic nuclei to the hippocampal formation and shows substantial levels of deposited synaptophysin in this white matter tract in ME7 animals with respect to NBH animals.


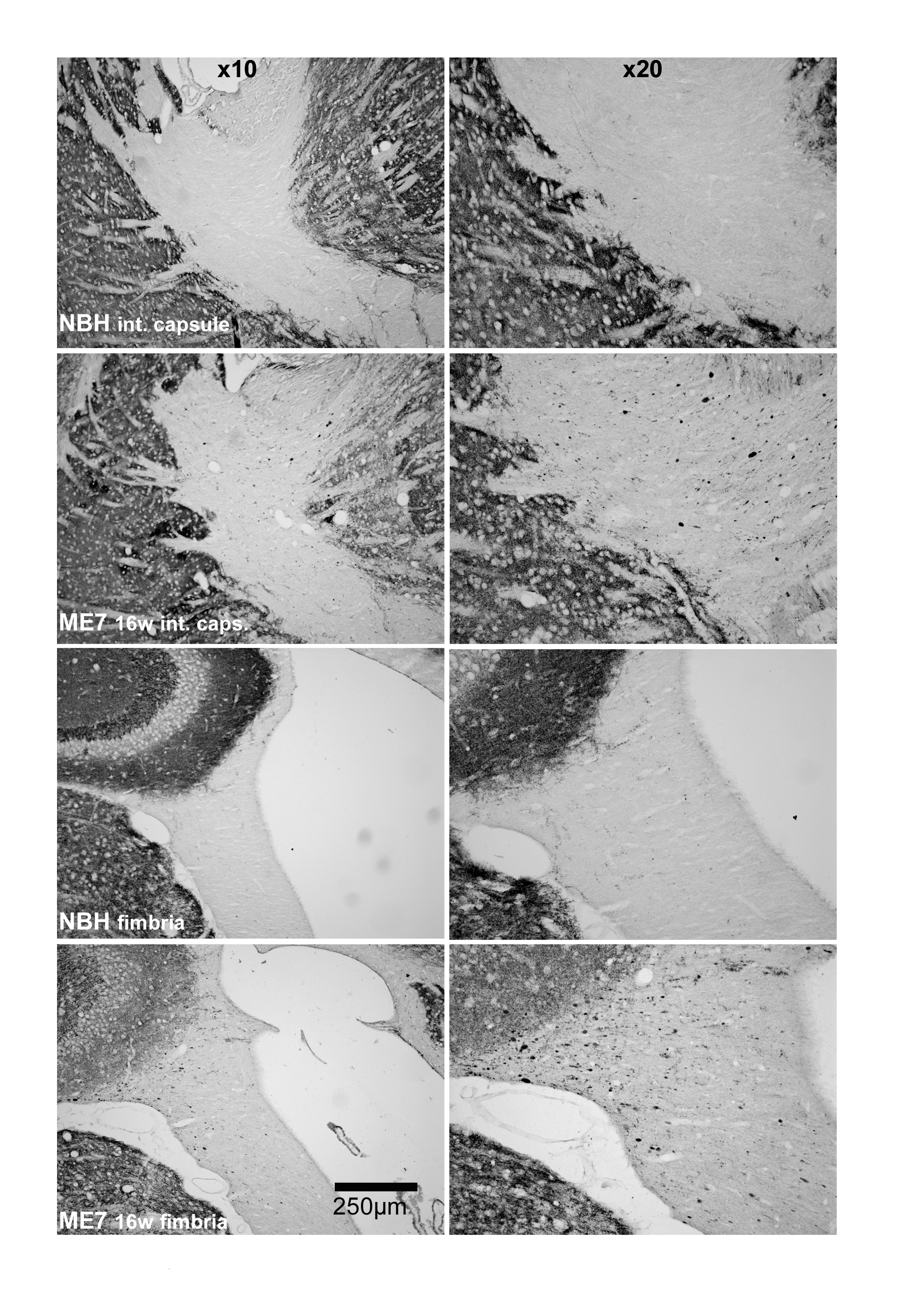


**Supplementary Figure 2.** Synaptophysin-labelled coronal brain sections from the normal (NBH) and diseased (ME7) brain at the level of the internal capsule (thalamus to cortex) and the fimbria (medial septum to hippocampus) white matter tracts. Sections were photographed with the 10x (left column) and 20x (right column) objectives as indicated. Sy38-positive spheroids are clearly visible in ME7 animals in both white matter tracts but these are absent in NBH animals. Scale bar=250µm (125µm in right column).

**Supplementary table 1.** Description of the association between experimental variables and errors on T-maze test during challenge (3-7 hours).

|  | |  | **RR** | **LCI** | **UCI** | **p** |
| --- | --- | --- | --- | --- | --- | --- |
| *Age group* | | |  | |  |  |
|  | NBH | | [ref] | |  |  |
|  | ME7 12w | | 1.48 | 1.15 | 1.90 | <0.01 |
|  | ME7 16w | | 2.33 | 1.87 | 2.91 | <0.01 |
|  | | |  |  |  |  |
| *Challenge* | | |  |  |  |  |
|  | Saline | | [ref] | |  |  |
|  | LPS | | 2.01 | 1.72 | 2.36 | <0.01 |
|  |  | |  |  |  |  |
| *Time point* | | |  |  |  |  |
|  | baseline | | [ref] | |  |  |
|  | 3 hrs | | 1.58 | 1.27 | 1.97 | <0.01 |
|  | 5 hrs | | 1.49 | 1.20 | 1.87 | <0.01 |
|  | 7 hrs | | 1.39 | 1.10 | 1.74 | 0.01 |
| *2-tailed p-value used in testing the null hypothesis that the coefficient (parameter) is 0, Wald χ2 for 6 d.f. = 156.93. | | | | | | |

**Supplementary table 2.** Testing the interaction between challenge and disease stage.

Question: Does the effect of challenge type vary in the different disease stage groups?

|  | | |  | **RR** | **LCI** | **UCI** | **p** |
| --- | --- | --- | --- | --- | --- | --- | --- |
| *Challenge x Age group* | | | |  | |  |  |
|  | Saline | NBH | | [ref] | |  |  |
|  | Saline | ME7 12w | | 1.11 | 0.70 | 1.75 | 0.66 |
|  | Saline | ME7 16w | | 2.11 | 1.43 | 3.13 | <0.01 |
|  |  |  | |  |  |  |  |
|  | LPS | NBH | | 1.68 | 1.09 | 2.59 | 0.02 |
|  | LPS | ME7 12w | | 2.81 | 1.88 | 4.19 | <0.01 |
|  | LPS | ME7 16w | | 4.06 | 2.79 | 5.92 | <0.01 |
|  | | | |  |  |  |  |
| *Time point* | | | |  |  |  |  |
|  | baseline | | | [ref] | |  |  |
|  | 3 hrs | | | 1.58 | 1.27 | 1.97 | <0.01 |
|  | 5 hrs | | | 1.49 | 1.20 | 1.87 | <0.01 |
|  | 7 hrs | | | 1.39 | 1.10 | 1.74 | 0.01 |
| *2-tailed p-value used in testing the null hypothesis that the coefficient (parameter) is 0, Wald χ2 for 8 d.f. = 157.20.  Test of challenge x age group interaction p<0.001. | | | | | | | |

Answer:

- ME7 16w mice perform worse than NBH, even when challenged with only saline
- LPS is associated with more errors in progressively more advanced ME7 mice. Note too that LPS is associated with worse cognition from baseline even in NBH mice (RR error 1.68, p=0.02).

**Supplementary table 3.** Testing the interaction between disease stage group and time course (during challenge) for errors in T-maze.

Question: Does the time course for errors vary in different age groups?

|  | |  | |  | **RR** | **LCI** | **UCI** | **p** |
| --- | --- | --- | --- | --- | --- | --- | --- | --- |
| *Age group x time point* | | | | |  |  |  |  |
|  | NBH | | baseline | | [ref] | |  |  |
|  | NBH | | 3 hrs | | 1.09 | 0.65 | 1.83 | 0.74 |
|  | NBH | | 5 hrs | | 0.76 | 0.43 | 1.35 | 0.35 |
|  | NBH | | 7 hrs | | 0.62 | 0.34 | 1.13 | 0.12 |
|  |  | |  | |  |  |  |  |
|  | ME7 12w | | baseline | | 0.89 | 0.53 | 1.50 | 0.66 |
|  | ME7 12w | | 3 hrs | | 1.75 | 1.11 | 2.76 | 0.02 |
|  | ME7 12w | | 5 hrs | | 1.22 | 0.75 | 1.98 | 0.43 |
|  | ME7 12w | | 7 hrs | | 1.28 | 0.79 | 2.07 | 0.32 |
|  |  | |  | |  |  |  |  |
|  | ME7 16w | | baseline | | 1.33 | 0.86 | 2.06 | 0.21 |
|  | ME7 16w | | 3 hrs | | 2.14 | 1.41 | 3.25 | <0.01 |
|  | ME7 16w | | 5 hrs | | 2.42 | 1.60 | 3.65 | <0.01 |
|  | ME7 16w | | 7 hrs | | 2.20 | 1.45 | 3.33 | <0.01 |
|  |  | |  | |  |  |  |  |
| *Challenge* | | | | |  | |  |  |
|  | Saline | | | | [ref] | |  |  |
|  | LPS | | | | 2.01 | 1.72 | 2.36 | <0.01 |
| *2-tailed p-value used in testing the null hypothesis that the coefficient (parameter) is 0, Wald χ2 for 12 d.f. = 169.68.  Test of time course x age group interaction p<0.001 | | | | | | | | |

Answer:

- The impairment is longest and most profound in ME7 16w mice (significant at all 3 time points)
- Most errors occurred in ME7 12w mice at 3 hours (RR 1.75, p=0.02)

**Supplementary table 4.** Description of the association between experimental variables and errors on T-maze test during recovery (22-24 hours).

|  | **RR** | **LCI** | **UCI** | **p** |
| --- | --- | --- | --- | --- |
| *Age group* |  | |  |  |
| NBH | [ref] | |  |  |
| ME7 12w | 1.03 | 0.76 | 1.41 | 0.84 |
| ME7 16w | 1.67 | 1.28 | 2.18 | <0.01 |
|  |  |  |  |  |
| *Challenge* |  | |  |  |
| Saline | [ref] | |  |  |
| LPS | 1.15 | 0.94 | 1.39 | 0.18 |
|  |  |  |  |  |
| *Time point* |  |  |  |  |
| baseline | [ref] | |  |  |
| 22 hrs | 1.21 | 0.96 | 1.53 | 0.11 |
| 24 hrs | 0.93 | 0.72 | 1.19 | 0.55 |
| *2-tailed p-value used in testing the null hypothesis that the coefficient (parameter) is 0, Wald χ2 for 5 d.f. = 30.70 | | | | |

- From this table, the challenge type (by itself) is not associated with errors during the recovery period.

**Supplementary table 5.** Testing the interaction between age group and time course (in recovery).

Question: Does the recovery from challenge vary between age groups?

|  | |  | |  | **RR** | **LCI** | **UCI** | **p** |
| --- | --- | --- | --- | --- | --- | --- | --- | --- |
| *Age group x time point* | | | | |  |  |  |  |
|  | NBH | | baseline | | [ref] | |  |  |
|  | NBH | | 22 hrs | | 1.00 | 0.59 | 1.70 | 1.00 |
|  | NBH | | 24 hrs | | 0.58 | 0.31 | 1.08 | 0.09 |
|  |  | |  | |  |  |  |  |
|  | ME7 12w | | baseline | | 0.86 | 0.51 | 1.45 | 0.57 |
|  | ME7 12w | | 22 hrs | | 1.08 | 0.66 | 1.78 | 0.75 |
|  | ME7 12w | | 24 hrs | | 0.72 | 0.42 | 1.25 | 0.25 |
|  |  | |  | |  |  |  |  |
|  | ME7 16w | | baseline | | 1.29 | 0.83 | 2.00 | 0.26 |
|  | ME7 16w | | 22 hrs | | 1.63 | 1.07 | 2.50 | 0.02 |
|  | ME7 16w | | 24 hrs | | 1.40 | 0.91 | 2.16 | 0.13 |
|  | | | | |  |  |  |  |
| *Challenge* | | | | |  |  |  |  |
|  | Saline | | | | [ref] | |  |  |
|  | LPS | | | | 1.15 | 0.94 | 1.39 | 0.18 |
| *2-tailed p-value used in testing the null hypothesis that the coefficient (parameter) is 0, Wald χ2 for 9 d.f. = 32.46. | | | | | | | | |

Answer

- There is lag in recovery in ME7 16w, where performance remains impaired at 22 hours (RR 1.62, p=0.02)

**References**

1. Cunningham C, Deacon RM, Chan K, Boche D, Rawlins JN, et al. (2005) Neuropathologically distinct prion strains give rise to similar temporal profiles of behavioral deficits. Neurobiol Dis 18: 258-269.

2. Murray C, Sanderson DJ, Barkus C, Deacon RM, Rawlins JN, et al. (2012) Systemic inflammation induces acute working memory deficits in the primed brain: relevance for delirium. Neurobiol Aging 33: 603-616 e603.

3. Folstein MF, Folstein SE, McHugh PR (1975) "Mini-mental state". A practical method for grading the cognitive state of patients for the clinician. J Psychiatr Res 12: 189-198.

4. Chatfield M, Matthews FE, Brayne C (2007) Using the Mini-Mental State Examination for tracking cognition in the older population based on longitudinal data. J Am Geriatr Soc 55: 1066-1071.

5. Charlson ME, Pompei P, Ales KL, MacKenzie CR (1987) A new method of classifying prognostic comorbidity in longitudinal studies: development and validation. J Chronic Dis 40: 373-383.
